# Disturbances in Brain Physiology Due to Season Play: A Multi-Sport Study of Male and Female University Athletes

**DOI:** 10.3389/fphys.2021.653603

**Published:** 2021-03-30

**Authors:** Nathan W. Churchill, Michael G. Hutchison, Simon J. Graham, Tom A. Schweizer

**Affiliations:** ^1^Keenan Research Centre for Biomedical Science of St. Michael’s Hospital, Toronto, ON, Canada; ^2^Neuroscience Research Program, St. Michael’s Hospital, Toronto, ON, Canada; ^3^Faculty of Kinesiology and Physical Education, University of Toronto, Toronto, ON, Canada; ^4^Department of Medical Biophysics, University of Toronto, Toronto, ON, Canada; ^5^Physical Sciences Platform, Sunnybrook Research Institute, Sunnybrook Health Sciences Center, Toronto, ON, Canada; ^6^Faculty of Medicine (Neurosurgery), University of Toronto, Toronto, ON, Canada; ^7^The Institute of Biomaterials & Biomedical Engineering (IBBME) at the University of Toronto, Toronto, ON, Canada

**Keywords:** exercise, season play, cerebral blood flow, white matter, brain activity

## Abstract

High-performance university athletes experience frequent exertion, resulting in disrupted biological homeostasis, but it is unclear to what extent brain physiology is affected. We examined whether athletes without overtraining symptoms show signs of increased neurophysiological stress over the course of a single athletic season, and whether the effects are modified by demographic factors of age, sex and concussion history, and sport-related factors of contact exposure and season length. Fifty-three university-level athletes were recruited from multiple sports at a single institution and followed longitudinally from beginning of season (BOS) to end of season (EOS) and 1 month afterwards, with a subset followed up at the subsequent beginning of season. MRI was used to comprehensively assess white matter (WM) diffusivity, cerebral blood flow (CBF), and brain activity, while overtraining symptoms were assessed with Hooper’s Index (HI). Although athletes did not report increased HI scores, they showed significantly increased white matter diffusivity and decreased CBF at EOS and 1 month afterwards, with recovery at follow-up. Global brain activity was not significantly altered though, highlighting the ability of the brain to adapt to exercise-related stressors. Male athletes had greater white matter diffusivity at EOS, but female athletes had greater declines in CBF at 1 month afterwards. Post-season changes in MRI measures were not related to change in HI score, age, concussion history, contact exposure, or length of athletic season. Hence, the brain shows substantial but reversible neurophysiological changes due to season play in the absence of overtraining symptoms, with effects that are sex-dependent but otherwise insensitive to demographic variations. These findings provide new insights into the effects of training and competitive play on brain health.

## Introduction

The benefits of a regular exercise regimen are well-established and include substantial improvements to mental health and cognitive function (Ruegsegger and Booth, 2018). These benefits are driven by multifactorial changes in brain physiology, as greater aerobic fitness is associated with reduced neuroinflammation (Svensson et al., 2015), greater resistance to oxidative stress (Radak et al., 2008), enhanced cerebral blood flow (CBF) and brain activity (Ainslie et al., 2008; Brown et al., 2010; Guiney et al., 2015), which are accompanied by improved performance on behavioral tasks (Erickson et al., 2015). Structural brain plasticity has also been observed, including greater gray matter (GM) volume and a greater density of white matter (WM) connections (Marks et al., 2007; Voss et al., 2013; Erickson et al., 2014; Herting et al., 2014). Regular exercise shows benefits across the aging span and may help to stave off age-related brain atrophy and functional declines (Cotman and Engesser-Cesar, 2002; Navarro et al., 2004).

Such changes in brain health represent long-term adaptations to exercise-related stress, which may be physiological and/or psychological in nature. During exercise, these stresses include increases in neurometabolism and neural activity, with consequently elevated CBF (Querido and Sheel, 2007; Ogoh and Ainslie, 2009). During post-exercise recovery, there are also acute reductions in neurometabolism and CBF, along with tissue edema, which are partly driven by oxidative stress and increased blood-brain barrier permeability (Poulin et al., 1999; Ayus et al., 2000; Ogoh and Ainslie, 2009; Bailey et al., 2011). Less is known about the long-term physiological brain changes that occur under chronic, sustained exertion. This is a fundamental part of high-performance athletics, however, which aims to optimize adaptive changes. For these athletes, a season may routinely involve 4–6 h of high-intensity training 5–6 days a week, interspersed with bouts of competition (Smith, 2003; Purvis et al., 2010; Carfagno and Hendrix, 2014). Regular high-intensity training can cause lasting disturbances in systemic homeostasis, including oxidative stress, inflammation, glycogen depletion, and autonomic imbalance (Tiidus, 1998; Meeusen et al., 2006; Tanskanen et al., 2010). These physiological loading effects may be substantial, even among athletes that do not show overt signs of overtraining, such as mood disturbances and self-reported fatigue (Purvis et al., 2010; Carfagno and Hendrix, 2014).

At present, there is an incomplete picture of how systemic changes occurring over a single athletic season affect cerebral physiology, and whether they represent reversible stress responses or persistent adaptive changes. It is also unclear to what extent such changes are influenced by key demographic factors of age, sex and concussion history (Lenroot and Giedd, 2006; Cosgrove et al., 2007; Churchill et al., 2017a), along with sport-related factors such as risk of contact exposure (Churchill et al., 2017b) and duration of the athletic season. To date, the literature has mainly focused on biomarkers and symptom assessments (Kreher and Schwartz, 2012; Meeusen et al., 2013), which only indirectly reflect neurophysiological stress. To address these knowledge gaps, the present study examined longitudinal brain changes for high-performance university athletes, drawn from a mixed cohort of male and female athletes in different sports, to identify generalizable effects of season play. MRI was used to comprehensively assess changes in WM microstructure, CBF and brain activity, and symptoms of overtraining were also monitored. Statistical testing was conducted to assess for longitudinal changes in MRI measures of brain physiology and overtraining symptoms. Supplemental testing examined the correlations of longitudinal changes in MRI measures with demographic and sport-related covariates.

## Materials and Methods

### Study Participants

Fifty three varsity athletes were recruited consecutively from university-level sport teams (volleyball, hockey, soccer, football, rugby, basketball, and lacrosse) at a single institution through the academic sport medicine clinic. All study participants were required to be free of neurologic impairments and to be fully recovered clinically from any prior concussions. Imaging was conducted at the beginning of season (BOS), end of season (EOS), and 1 month after EOS (1MO), with a subset of athletes receiving follow-up imaging at the subsequent beginning of season (BOS2). Within the longitudinal study, some athletes had missed imaging sessions. The number of participants retained at each time point was: BOS (53/53), EOS (42/53), 1MO (41/53), and BOS2 (19/53). For the BOS2 scans, 18 were acquired at one year post-BOS and one was acquired at two years post-BOS. The latter datapoint was retained after verifying it did not significantly alter the main analysis results. Attrition was not significantly related to demographic variables (age, sex, concussion history, contact exposure, and season length), based on Spearman correlations at a False Discovery Rate (FDR) threshold of 0.05. The study was carried out in accordance with the Canadian Tri-Council Policy Statement 2 and approval of University of Toronto and St. Michael’s Hospital research ethics boards, with all participants giving free and written informed consent. The datasets analyzed for this study can be found in the *figshare* repository at https://figshare.com/s/60c2119945b834a17465, and anonymized imaging data and code will be shared upon request from any qualified investigator.

### Magnetic Resonance Imaging

Athletes were imaged using a 3 Tesla MRI system (Magnetom Skyra) with standard multi-channel head coil. Structural imaging included: three-dimensional T1-weighted Magnetization Prepared Rapid Acquisition Gradient Echo imaging [MPRAGE: inversion time (TI)/echo time (TE)/repetition time (TR) = 1,090/3.55/2,300 ms, flip angle (θ) = 8°, 192 sagittal slices with field of view (FOV) = 240 mm × 240 mm, 256 × 256 pixel matrix, 0.9 mm slice thickness, 0.9 mm × 0.9 mm in-plane resolution, with bandwidth (BW) = 200 Hz per pixel (Hz/px)], fluid attenuated inversion recovery imaging (FLAIR: TI/TE/TR = 1,800/387/5,000 ms, 160 sagittal slices with FOV = 230 mm × 230 mm, 512 × 512 matrix, 0.9 mm slice thickness, 0.4 mm × 0.4 mm in-plane resolution, and BW = 751 Hz/px), and susceptibility-weighted imaging (SWI: TE/TR = 20/28 ms, θ = 15°, 112 axial slices with FOV = 193 mm × 220 mm, 336 × 384 matrix, 1.2 mm slice thickness, 0.6 mm ×0.6 mm in-plane resolution, and BW = 120 Hz/px). The MPRAGE, FLAIR, and SWI scans were inspected by an MRI technologist during imaging and later reviewed by a neuroradiologist, with clinical reporting if abnormalities were identified. No abnormalities (white matter hyper-intensities, contusions, micro-hemorrhage, or statistical outliers) were found for the athletes in this study.

Advanced MRI sequences were also acquired, including diffusion tensor imaging (DTI) to assess brain tissue microstructure. This was evaluated in terms of mean diffusivity (MD), characterizing the average rate of water diffusion and fractional anisotropy (FA), characterizing the directionality of water diffusion; this study focused on MD within white matter tracts but FA results are shown in Supplementary File 1 for completeness. Voxel-wise estimates of CBF were obtained in units of ml/100 g/min using arterial spin labeling (ASL). Resting brain activity was also indexed using blood-oxygenation level dependent functional MRI (fMRI; BOLD fMRI), by calculating the fractional amplitude of low frequency fluctuations (FALFF). Full details of the imaging sequences and data processing are described below, where processing and analysis were performed using the fMRIB Software Library (FSL; https://fsl.fmrib.ox.ac.uk/fsl/fslwiki), DTI ToolKit (DTI-TK; http://dti-tk.sourceforge.net/), Analysis of Functional Neuroimages (AFNI; afni.nimh.nih.gov), and customized algorithms developed in the laboratory.

#### Diffusion Tensor Imaging

A DTI protocol was performed (66 axial slices with FOV = 240 mm × 240 mm, 120 × 120 matrix, 2.0 mm slice thickness, 2.0 × 2.0 in-plane resolution, and BW = 1,736 Hz/Px), consisting of 30 diffusion-weighting directions (TE/TR = 83/7,800 ms, *b* = 700 s/mm^2^, with nine b0 scans). The data were processed using FSL utilities and custom software. The *eddy* protocol was used to perform simultaneous correction of eddy currents and rigid-body head motion, *bet* was used to mask out non-brain voxels, and *dtifit* used to calculate voxel-wise measures of FA and MD. Co-registration of DTI maps to a common template was obtained using DTI-TK software with default parameter settings.^1^ The IXI Aging DTI Template 3.0 was used as an initial reference and a subset of 20 independent athlete scans were selected to generate a longitudinally unbiased athlete template (five from each of BOS, EOS, 1MO, and BOS2). For this subset, a bootstrapped template was obtained using *dti_template_bootstrap*, with affine alignment and template updating using *dti_affine_population* (three iterations), then diffeomorphic alignment and template updating using *dti_diffeomorphic_population* (six iterations). For all remaining athletes, transforms to the athlete group template were obtained by sequentially applying rigid (*dti_rigid_reg*), affine (*dti_affine_reg*), and diffeomorphic (*dti_diffeomorphic_reg*) registration steps. The transform of the athlete template into MNI space was then obtained using the IIT Human Brain Atlas’ mean tensor template as a reference, with sequential application of rigid (*dti_rigid_reg*), affine (*dti_affine_reg*), and diffeomorphic (*dti_diffeomorphic_reg*) registration steps. Afterward, the net transforms from subject space to MNI space were computed using *dfRightComposeAffine* and were applied to all DTI parameter maps *via deformationScalarVolume*. During registration, images were resampled to 3 mm isotropic voxel resolution, and a 6 mm full width at half-maximum (FWHM) 3D Gaussian smoothing kernel applied to reduce spatial noise. Analysis was performed within a mask of white matter regions, i.e., where FA > 0.30 in the group template, with manual segmentation and exclusion of brain stem areas with substantial field inhomogeneity.

#### Arterial Spin Labeling

2D pulsed ASL was acquired using the PICORE QUIPSS II sequence (TE/TR = 12/2,500 ms, TI1/TI1s/TI2 = 700/1,600/1,800 ms, θ = 90°, 14 oblique-axial slices with FOV = 256 mm × 256 mm, 64 × 64 matrix, 8.0 mm slice thickness with 2.0 mm gap, 4.0 mm ×4.0 mm in-plane resolution, and BW = 2,368 Hz/px). A single calibration image was acquired to estimate the equilibrium magnetization M_0_, together with a series of 45 tag-control image pairs. The data were processed using AFNI utilities and custom software. Rigid-body motion correction of tag-control image pairs was performed using *3dvolreg* to align to the M_0_ image. Filtering of outlier tag-control pairs was performed using an established protocol (Tan et al., 2009), followed by spatial smoothing with *3dmerge* using a 3D Gaussian kernel with 6 mm isotropic FWHM. Voxel-wise estimates of CBF were calculated in units of ml/100 g/min based on the mean difference of all tag-control pairs, using established kinetic modeling parameters (Churchill et al., 2017c). The CBF maps were co-registered using FSL software.^2^ The MNI152 template was used as reference and *flirt* used to compute the rigid-body alignment of the mean ASL volume to the T1 image, and the affine alignment of the T1 image to the MNI template. The net transforms from subject space to MNI space were computed using *convert_xfm* and applied to all CBF maps *via flirt*, with resampling at 3 mm isotropic voxel resolution. To ensure that only gray matter regions were analyzed, voxels were retained that intersected with the MNI152 brain mask and a gray matter mask. The latter was obtained by applying *fast* to participant T1 images, producing segmented GM, WM, and cerebrospinal fluid (CSF) maps. The maps were then aligned to the MNI152 template using the *fslvbm* protocol, resampled to 3 mm isotropic voxel resolution and smoothed using a 3D Gaussian kernel with 6 mm isotropic FWHM, followed by group averaging. A gray matter mask was then chosen to include only voxels with probability values *p*(GM) > *p*(WM) + *p*(CSF). Remaining voxels that overlapped with ventricles of the template were removed manually. To further control against white matter partial volume effects, an additional masking step was performed, retaining only voxels with mean baseline CBF values >20 ml/100 g/min.

#### Functional MRI

Resting-state fMRI was acquired *via* multi-slice T2∗-weighted echo planar imaging (EPI: TE/TR = 30/2,000 ms, θ = 70°, 32 oblique-axial slices with FOV = 200 mm × 200 mm, 64 × 64 matrix, 4.0 mm slice thickness with 0.5 mm gap, 3.125 mm × 3.125 mm in-plane resolution, and BW = 2,298 Hz/px), producing a time-series of 195 images. During acquisition, athletes were instructed to lie still with their eyes closed and to not focus on anything. The data were processed using AFNI utilities and custom software. After discarding the first four volumes to allow scans to reach equilibrium, this included rigid-body motion correction with *3dvolreg*, removal of outlier scan volumes using SPIKECOR,^3^ slice-timing correction with *3dTshift*, spatial smoothing with a 6 mm FWHM isotropic 3D Gaussian kernel *via* *3dmerge*, and regression of motion parameters and linear-quadratic trends as nuisance covariates. To control for physiological noise, PHYCAA+^4^ was used to spatially down-weight areas of non-neural signal, followed by regression of the signal from WM and CSF. The latter regressions were done after co-registering the fMRI data to a common MNI152 template using the same approach as described for the ASL data above, with resampling at 3 mm isotropic voxel resolution. Afterwards for WM, a mask of voxels in the 95th percentile of p(WM) values was obtained and a single spatial erosion performed (3×3 in-plane kernel). Two seed time series were obtained by averaging voxels within cerebral white matter and within brainstem white matter. For CSF, a mask of voxels in the 95th percentile of p(CSF) values was obtained. Two seed time series were obtained by averaging voxels within left and right lateral ventricles. The four physiologic time series were then regressed from the data. To ensure only gray matter regions were analyzed, voxels were then retained that intersected with the MNI152 brain mask and a gray matter mask, using the procedure described for the ASL data above. After co-registration, the fractional amplitude of low-frequency fluctuations (FALFF) was calculated as follows. At each voxel, the BOLD power spectral density was computed using a Welch estimator (Hanning window, 50% overlap), followed by summing spectral power over the interval of 0.015–0.08 Hz, and then dividing by total spectral power. This provides a standardized estimate of regional brain activity.

### Participant Demographics and Clinical Data

The demographics for athletes are reported in Table 1, including age, sex, and history of concussion (HOC). Data are also reported for Hooper’s Index (HI), which is a sensitive tool for monitoring overtraining and recovery in athletes, and has been validated against systemic biomarkers, including red and white blood cell counts, catecholamines, and creatine kinase (Hooper and Mackinnon, 1995; Hooper et al., 1999). The HI uses a seven-point Likert scale to assess how athletes felt with respect to eight questions probing fatigue, stress, sleep, muscle soreness, irritability, and general health. Summed over all items, a higher HI score reflected higher levels of stress and lower perceived well-being. Unless otherwise noted, group statistics are summarized by the median and interquartile range (Q1, Q3). Differences relative to baseline were assessed for the HI score at each imaging session (EOS, 1MO, and BOS2) relative to BOS, using paired-measures Wilcoxon tests, with missing data handled *via* pairwise deletion. Other measures potentially influencing brain physiology were also assessed, including number of hours of sleep the previous night and time since the last bout of exercise. For female athletes, information was also collected about the use of hormonal contraceptives, with data collected from 27/29 at BOS and 22/29 following EOS. Sport statistics are reported in Table 2, including the sample size for each sport, a contact exposure rating based on the Meehan et al. classification (Meehan III et al., 2016), where 1 = non-contact, 2 = limited contact, and 3 = collision, along with season length, given as the number of days from an athlete’s BOS scan to their EOS scan.

**Table 1:** Participant demographics, including age, sex, and history of concussion (HOC).

**Age** **(mean ± SD)**	19.9 ± 1.7 years			
**Female**	29/53 (55%)			
**HOC**	22/53 (42%)			
	BOS	EOS	1MO	BOS2
Hooper’s index (HI)	23 (18, 29)	25 (21, 31)	27 (21, 30)	25 (19, 31)
Hours of sleep (h)	7 (6, 8)	8 (7, 8)	7 (6, 8)	7 (6, 8)
Time since activity (h)	15 (6, 24)	24 (10, 48)	22 (16, 26)	13 (3, 15)

Other measures include Hooper’s Index (HI), which summarizes overtraining symptoms, hours of sleep the night before testing, and time since last physical activity. These measures are summarized by the median and interquartile range (Q1, Q3).

**Table 2:** Participant sport information, including the number of athletes participating in each sport (*N*), the sport’s contact exposure rating (1 = non-contact, 2 = limited contact, and 3 = collision) and season length, given as the number of days from BOS scan to EOS scan, summarized by the median and (minimum, maximum) for all participants in each sport.

	Male athletes			Female athletes		
	*N*	Contact exposure	Season length (dy)	*N*	Contact exposure	Season length (dy)
Volleyball	3	1	155 (145, 181)	5	1	145 (138, 160)
Hockey	8	3	142 (131, 151)	13	2	151 (56, 176)
Soccer	2	2	66 (63, 68)	3	2	70 (70, 77)
Football	4	3	68 (57, 70)	0	–	–
Rugby	1	3	47	1	3	117
Basketball	2	2	149 (146, 151)	5	2	140 (120, 168)
Lacrosse	4	3	56 (55, 70)	2	2	57 (52, 62)

### Effects of Season Play

For the MRI data, global MD scores (gMD) were calculated by averaging over all white matter voxels, while global CBF (gCBF) and global FALFF (gFALFF) scores were calculated by averaging over all gray matter voxels. To estimate longitudinal change in global scores in the presence of missing data (see Materials and Methods: Study Participants), the effect of imaging session on global response was estimated within a linear mixed effects model (LMM), with fixed effects of imaging session (EOS, 1MO, and BOS2) measured relative to BOS. The models also included fixed effect covariates adjusting for demographic factors of age, sex, and HOC, along with participant-specific random-effects intercepts. The models were fitted using the Matlab R2017b *fitlme* package (The MathWorks, Natick, MA) in a bootstrap resampling framework, where resampling units consisted of all measures for a given participant (1,000 iterations). This produced fixed-effect coefficients *b* with 95%CIs, bootstrap ratios (BSRs; mean divided by bootstrap standard error) and *p* values. Significant sessions were identified by thresholding at an FDR of 0.05. To visualize the spatial patterns of affected brain regions, analysis of longitudinal change was also conducted at the voxel level by performing bootstrap analysis of the paired-measures differences (EOS-BOS, 1MO-BOS, and BOS2-BOS), with missing data handled *via* pairwise deletion. Brain maps of standardized effect sizes were then displayed in terms of the *z*-distributed BSR values, which were thresholded at a nominal |BSR| > 2 (approximately *p* < 0.05, uncorrected).

### Demographic and Sport-Related Covariates

Given the limited prior investigation of multi-parameter brain changes associated with season play, secondary analyses explored the associations between longitudinal changes in global MRI measures and multiple covariates of interest. The paired-measures MRI change scores (EOS-BOS, 1MO-BOS, and BOS2-BOS) were first correlated with the corresponding paired-measures HI change scores, to determine whether MRI effects were related to worsening symptoms of over-training. The MRI change scores were then correlated with demographic factors of age, sex and HOC, all of which may substantially affect neurophysiology (Lenroot and Giedd, 2006; Cosgrove et al., 2007; Churchill et al., 2017a). The MRI change scores were further correlated with sport-related measures of contact exposure rating and individual athletes’ season length, both defined in Participant Demographics and Clinical Data above. All analyses were conducted *via* Spearman rank correlation with pairwise deletion of missing data; bootstrapping (1,000 iterations) was used to obtain 95%CIs, BSRs, and *p* values. Significant effects were identified across sessions and MRI parameters, at an FDR of 0.05. For significant sex-related effects, secondary analyses also tested for correlations between the MRI change scores and the use of hormonal contraceptives among female athletes.

## Results

### Participant Demographics

As shown in Tables 1, 2, study participants included a mixture of male and female athletes with and without prior HOC, drawn from a variety of different sports. For athletes with HOC, the most recent concussion occurred a median of 35 (19, 58) months prior to baseline imaging. The athletes showed moderate HI scores that were comparable across imaging sessions, with no significant changes from BOS to later sessions (|*z*| ≤ 1.48 and *p* ≥ 0.138, for all comparisons). Similarly, there were no significant differences between imaging sessions, in number of hours sleep the previous night (|*z*| ≤ 1.01 and *p* ≥ 0.092, for all comparisons). Relative to BOS, the average time since physical activity was slightly longer for EOS (*z* = 2.85, *p* = 0.004) and 1MO (*z* = 2.25, *p* = 0.025) but not for BOS2 (*z* = −0.29, *p* = 0.767); athletes had exercised on average within 24 h of assessment for all imaging sessions. Among the female athletes reporting on hormonal contraceptive use, 13/27 (48%) were using contraceptives at BOS, with three non-users becoming users post-EOS and one user becoming a non-user post-EOS.

### Effects of Season Play

**Figure 1:**
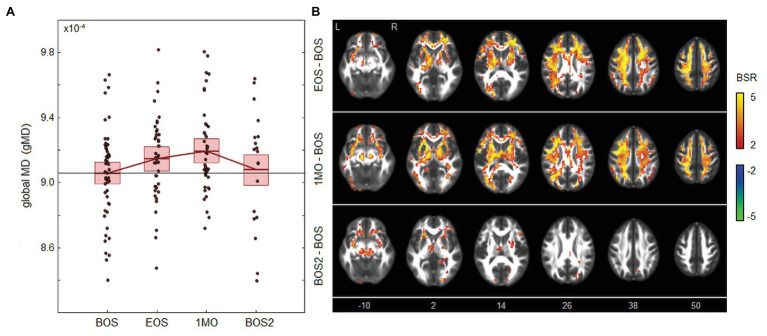
Effects of season play on white matter mean diffusivity (MD). **(A)** The distribution of athlete global MD (gMD) values, plotted for each imaging session, including beginning of season (BOS), end of season (EOS), one month afterwards (1MO), and follow-up beginning of season (BOS2). The horizontal red lines denote group means, and boxes indicate 95% CIs of the mean; distribution means are connected between sessions by solid red lines; the mean BOS value is also plotted as a horizontal black line as a reference. **(B)** Maps of regional change in MD, with relative effect sizes given as *z*-distributed bootstrap ratio (BSR) values, thresholded at |BSR| > 2 (approximately *p* < 0.05, uncorrected). The *z*-axis coordinates of axial slices in MNI space are also shown.

As depicted in Figure 1, there were changes in global white matter diffusivity associated with season play. Figure 1A depicts significantly elevated mean gMD values at EOS [mean and 95%CI: 0.97 × 10^−5^, (0.58, 1.39) × 10^−5^, BSR = 4.90, *p* < 0.001] and 1MO [1.34 × 10^−5^, (0.87, 1.81) × 10^−5^, BSR = 5.68, *p* < 0.001] at an FDR of 0.05, returning to near-baseline at BOS2 [0.26 × 10^−5^, (−0.75, 1.22) × 10^−5^, BSR = 0.52, *p* = 0.594]. Brain maps in Figure 1B show changes that are distributed throughout the brain at EOS, with greatest effects in anterior and dorsal white matter of the corona radiata. Effect sizes appear to be similar in magnitude at 1MO but are more spatially extensive, including greater posterior white matter effects. At BOS2, the changes relative to BOS have largely dissipated, except for sparse effects seen mainly in the external capsule and cerebral peduncle.

**Figure 2:**
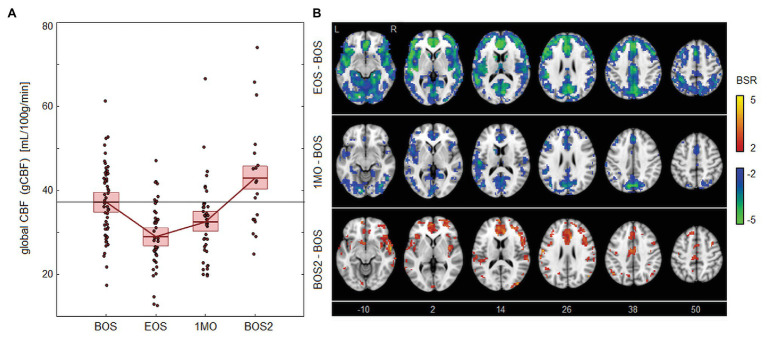
Effects of season play on cerebral blood flow (CBF). **(A)** The distribution of athlete global CBF (gCBF) values, plotted for each imaging session, including beginning of season (BOS), end of season (EOS), one month afterwards (1MO), and follow-up beginning of season (BOS2). The horizontal red lines denote group means, and boxes indicate 95% CIs of the mean; distribution means are connected between sessions by solid red lines; the mean BOS value is also plotted as a horizontal black line as a reference. **(B)** Maps of regional change in CBF, with relative effect sizes given as *z*-distributed BSR values, thresholded at |BSR| > 2 (approximately *p* < 0.05, uncorrected). The *z*-axis coordinates of axial slices in MNI space are also shown.

As seen in Figure 2, there were also declines in blood flow associated with season play. Figure 2A shows that CBF is significantly reduced at EOS [−8.02, (−10.39, −5.61) ml/100 g/min, BSR = −6.55, *p* < 0.001] and 1MO [−4.68, (−7.66, −1.43) ml/100 g/min, BSR = −2.99, *p* < 0.001] at an FDR of 0.05. The effect is then reversed at follow-up, with significantly elevated CBF at BOS2 compared to the previous baseline [5.84, (0.29, 11.73) ml/100 g/min, BSR = 2.05, *p* = 0.038]. Brain maps in Figure 2B show extensive changes at EOS, with greatest effects in the cerebellum, insula, cingulate cortex, and precuneus. At 1MO, a similar spatial pattern is seen, but effects have reduced in magnitude and spatial extent, except for the precuneus where effects are relatively unchanged. At BOS2, elevated CBF is seen anteriorly in the brain, including regions that were previously reduced at EOS, such as the insula and anterior cingulate.

**Figure 3:**
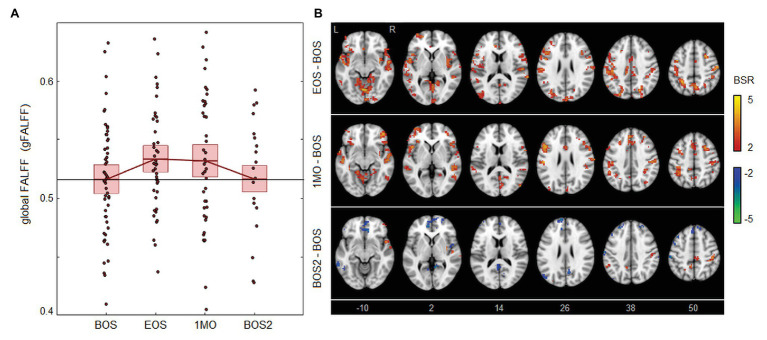
Effects of season play on brain activity, measured as the fractional amplitude of low-frequency fluctuations (FALFF). **(A)** The distribution of athlete global FALFF (gFALFF) values, plotted for each imaging session, including beginning of season (BOS), end of season (EOS), one month afterwards (1MO), and follow-up beginning of season (BOS2). The horizontal red lines denote group means, and boxes indicate 95% CIs of the mean; distribution means are connected between sessions by solid red lines; the mean BOS value is also plotted as a horizontal black line as a reference. **(B)** Maps of regional change in FALFF, with relative effect sizes given as *z*-distributed BSR values, thresholded at |BSR| > 2 (approximately *p* < 0.05, uncorrected). The *z*-axis coordinates of axial slices in MNI space are also shown.

As shown in Figure 3, the effects of season play on brain activity are far more modest than for the other parameters. The FALFF values seen in Figure 3A have slight but non-significant elevations at EOS [1.75 × 10^−2^, (0.05, 3.20) × 10^−2^, BSR = 1.97, *p* = 0.040] and 1MO [1.48 × 10^−2^, (−0.49, 3.20) × 10^−2^, BSR = 1.46, *p* = 0.154] at an FDR of 0.05, with further diminished effects at BOS2 [0.01 × 10^−2^, (−2.10, 2.21) × 10^−2^, BSR = −0.06, *p* = 0.924]. Brain maps in Figure 3B show a similarly scattered pattern of weakly elevated FALFF, with higher values seen particularly in the cerebellum and parietal cortex at EOS, and in orbitofrontal and inferior frontal regions at 1MO. At BOS2, the FALFF effects are sparser, with limited decreases in prefrontal areas and increases in postcentral and parietal regions.

### Demographic and Sport-Related Covariates

**Figure 4:**
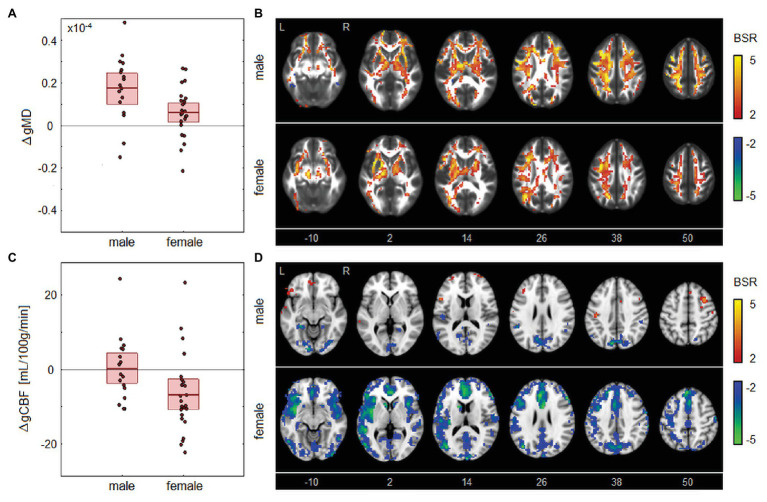
Longitudinal changes in MRI parameters showing significant associations with sex. **(A)** The distribution of longitudinal changes in global MD (gMD) from beginning of season (BOS) to end of season (EOS), plotted for male and female groups. The horizontal red lines denote group means, and boxes indicate 95% CIs of the mean. **(B)** Maps of regional change in MD for male and female groups, with relative effect sizes given as BSR values, thresholded at |BSR| > 2 (approximately *p* < 0.05, uncorrected). **(C)** The distribution of longitudinal changes in global CBF (gCBF) from BOS to one month post-season (1MO), plotted for male and female groups. The horizontal red lines denote group means and boxes indicate 95% CIs of the mean. **(D)** Maps of regional change in CBF for male and female groups, with relative effect sizes given as z-distributed BSR values, thresholded at |BSR| > 2. The *z*-axis coordinates of axial slices in MNI space are also shown.

There were no significant correlations between the longitudinal changes in global MRI parameters and corresponding changes in HI scores, for any of the imaging sessions (|*ρ*| ≤ 0.272 and *p* ≥ 0.190, for all tests). Among the demographic factors, correlations with MRI parameters were also non-significant for age (|*ρ*| ≤ 0.344 and *p* ≥ 0.134, for all tests) and for HOC (|*ρ*| ≤ 0.346 and *p* ≥ 0.142, for all tests). However, sex was significantly correlated with changes in MRI parameters, as depicted in Figure 4. This included gMD changes from BOS to EOS [*ρ* and 95%CI: −0.430, (−0.688, −0.126), BSR = −3.01, *p* = 0.004], indicating a greater increase in gMD for male athletes over this time interval, as seen in Figure 4A. Brain maps in Figure 4B depict similar patterns of increased diffusivity for males and females, but with greater effect sizes among males, particularly in posterior and dorsal corona radiata. Sex was also significantly correlated with gCBF changes from BOS to 1MO [−0.398, (−0.640, −0.112), BSR = −2.96, *p* = 0.007], indicating a greater decline in gCBF among female athletes over this time interval, as shown in Figure 4C. Brain maps in Figure 4D show that CBF changes are sparser for male athletes, with declines in the occipital cortex and cuneus, along with more limited increases in frontal regions. By contrast, female athletes show large declines throughout gray matter, with effects being most prominent anteriorly, within insular, anterior cingulate, and superior frontal regions. Both of the identified sex effects attained significance at an FDR of 0.05, with |*ρ*| ≤ 0.385, *p* ≥ 0.093 for all other tests. Among female athletes where contraceptive use did not change between BOS and EOS, use was non-significantly correlated with changes in gMD from BOS to EOS [−0.096 (−0.563, 0.414), BSR = −0.38, *p* = 0.696], and with changes in gCBF from BOS to 1MO [−0.094 (−0.659, 0.502), BSR = −0.32, *p* = 0.724]. Contact exposure rating was negatively correlated with gFALFF change from BOS to BOS2 [−0.456 (−0.771, −0.001), BSR = −2.31, *p* = 0.044] but was non-significant at an FDR of 0.05 and had |*ρ*| ≤ 0.199, *p* ≥ 0.390 for all other tests. Season length was also non-significantly correlated with changes in MRI parameters [|*ρ*| ≤ 0.431 and *p* ≥ 0.097, for all tests].

## Discussion

Regular exercise may confer substantial long-term brain health benefits, due to adaptive physiological stress responses (Cotman and Engesser-Cesar, 2002; Navarro et al., 2004). Less is known, however, about the impact of sustained, chronic exertion on athlete cerebral physiology during a season of play. This study addressed the literature gap by acquiring longitudinal, multi-parameter MRI data for a mixed cohort of university athletes. The key finding was evidence of substantial global disturbances in cerebral physiology in the absence of overtraining symptoms, emphasizing that the brain is highly sensitive to the effects of sport participation. These measures had recovered by the beginning of next season, however, denoting a lack of significant, long-lasting disruptions. There was also evidence of sex differences in the effects of season play on MRI parameters. Intriguingly, the observed effects did not vary significantly with age, concussion history, level of contact exposure, or length of athletic season.

The DTI analyses indicate significant changes in the microstructure of white matter at EOS and 1 month afterward. The observed increases in global MD are consistent with a neuroinflammatory response to training and sport participation, which may involve glial hypertrophy (Streit et al., 2004). In addition, an increase in circulating reactive oxygen species can induce blood-brain barrier permeability, leading to cerebral edema (Marmarou, 2007; Lochhead et al., 2010). Both mechanisms may increase extra-axonal water, thereby increasing diffusivity within white matter tissues. The MD parameter also shows evidence of delayed onset, with effects that are largest 1 month post-season, despite most athletes downscaling their training at this time. This may be due to self-reinforcing processes, e.g., neuroinflammation inducing blood-brain barrier permeability, which in turn increases neuroinflammatory response (Gurney et al., 2006; Marchi et al., 2013). Alternatively, it may indicate an ongoing depletion of antioxidant capabilities between these time points (Radak et al., 2008). Nevertheless, MD effects have normalized at the beginning of next season, indicating delayed but ultimately transient changes in cerebral physiology. Intriguingly, the demographic analyses show greater MD response for male athletes compared to females at EOS, but comparable values 1 month afterward. This may be due to sex differences in training load, or it may represent differences in the latency of neurophysiological response. For example, sex differences in microglial function have been identified, including greater reactivity among males (Yanguas-Casás, 2020), which may contribute to elevated MD in this group at EOS. Alternatively, estrogen has antioxidant capabilities (Joo et al., 2004), which may serve as a “buffer” that initially mitigates the effects of season play on MD among female athletes.

The ASL analyses also show a significant reduction in gCBF at the end of the season and 1 month afterward. The reductions in CBF are consistent with reduced blood-brain barrier integrity, mediated by increases in oxidative stress and neuroinflammatory response, which can impair microcirculation and reduce cerebral autoregulatory capacity (Wahl and Schilling, 1993; Uhl et al., 1994; Bailey et al., 2011). Compared to DTI, the effects appear to resolve more rapidly, with the greatest CBF reductions seen at EOS and partial normalization of CBF values occurring 1 month afterward. This underscores the highly reactive nature of the cerebrovasculature to physiological stressors, which has been documented in athletes in response to normal exertion (Thomas et al., 1989) and in pathological brain states such as concussion (Len and Neary, 2011). By the next BOS, however, CBF values have become significantly elevated relative to prior year baseline. The enhanced CBF at follow-up is consistent with body of literature showing long-term adaptive changes in blood flow due to exercise (Ainslie et al., 2008; Murrell et al., 2013). This suggests that, despite the initial CBF reductions, the training loads confer long-term cerebrovascular health benefits. Supplemental analyses show greater CBF declines for female athletes compared to males, seen 1 month after EOS. The presence of sex differences in CBF is unsurprising, given that sex hormones play a role in blood flow regulation (Armstead, 2016), and the results are consistent with evidence that female athletes have more reactive cerebrovascular systems compared to their male counterparts (Kastrup et al., 1997; Tallon et al., 2020).

In contrast to the reliable changes in tissue microstructure and CBF, analyses of BOLD fMRI show limited changes in global resting brain activity after season play, as measured with FALFF. This provides evidence that the changes in brain physiology, which include reduced CBF, do not cause substantial declines in neural function. In fact, regional analyses show modest increases in resting brain activity at end of season, possibly reflecting training-related increases in energy metabolism (Steiner et al., 2011). This maintenance of neural activity is consistent with an absence of significant self-reported functional impairments based on HI scores, and it highlights the ability of the brain to successfully adapt to physiological stressors. One potential concern, however, is that sustained neurometabolic activity in the presence of reductions in gCBF may lead to subtle increases in oxidative stress over time (Giza and Hovda, 2014). This is a potential contributor to the heightened MD effects that are seen at 1 month post-season.

Although this study has enhanced our understanding of the effects of season play on brain physiology, further work is needed to fully understand how these changes evolve over time and their underlying mechanisms. This study focused on testing for brain changes from beginning to end of season. It is now important to understand how they progress over the course of a season, i.e., whether effects increase throughout the season or whether they peak early in training and remain relatively static afterward. Given the lack of correlations with symptom reporting, future research should also examine associations with objective measures of training load, e.g., by monitoring the type, frequency, and intensity of exercises, which may vary significantly between sports and between individuals. Physiological indices of physical and psychological stress should also be examined, such as cortisol, creatine kinase, blood cytokines, and heart rate variability (Aubert et al., 2003; Robson-Ansley et al., 2007; Balsalobre-Fernández et al., 2014; Freire et al., 2020). Future studies should also account for cardiovascular factors that may be altered by season play, including arterial CO_2_ tension and blood pressure. Under normal conditions, regulatory mechanisms maintain consistent CBF despite fluctuations in these parameters (Willie et al., 2014). However, if the regulatory mechanisms are disrupted by chronic exertion, then CBF may be significantly affected, making it presently unclear to what extent they contribute to the observed post-season changes in blood flow. Given the intriguing sex differences identified, future research should also focus on replicating study findings and characterizing the mechanisms of sex differences in greater detail. While the present study found no evidence that hormonal contraceptive use influenced results, other factors, including phase of the menstrual cycle, contribute to fluctuations in female hormone levels. This may increase the variability of MRI measures, and potentially reduces the precision and accuracy of estimated sex differences in longitudinal brain changes. Lastly, this study was conducted entirely on a sample of high-performance university athletes. Future research should incorporate appropriate controls, e.g., university students at varying levels of physical fitness, to establish the relationships more definitively between MRI parameters, chronic exertion, and physical fitness.

This study provides evidence of substantial changes in brain physiology in response to season play, likely from the stress of repeated, exertional activities (i.e., training, practice, and competitive play). These effects generalized across demographic cohorts and were not, in general, related to issues such as age, concussion history, contact exposure, or duration of season play. The effects were also seen in the absence of self-reported symptoms of overtraining, indicating that sport-related stresses on brain physiology are not necessarily detected using standard assessment tools. These issues are of interest, particularly given that training objectives for high-performance athletes involve calibrating their training load to maximize performance and physiological adaptation. The study shows the promise of advanced MRI in detecting these brain changes and developing biomarkers to study optimal training regimens and long-term effects on brain health, along with the potential effects of overtraining.

## Data Availability Statement

The datasets presented in this study can be found in online repositories. The names of the repository/repositories and accession number(s) can be found below: the datasets analyzed for this study can be found in the figshare repository at https://figshare.com/s/60c2119945b834a17465, and anonymized imaging data and code will be shared upon request from any qualified investigator.

## Ethics Statement

The studies involving human participants were reviewed and approved by University of Toronto and St. Michael’s Hospital Research Ethics Boards. The patients/participants provided their written informed consent to participate in this study.

## Author Contributions

NC was involved in study design, analysis planning and execution, interpretation of results, and manuscript writing. MH and SG were involved in study design and critical revision of manuscript. TS was involved in study design, interpretation of results, and critical revision of manuscript. All authors contributed to the article and approved the submitted version.

### Conflict of Interest

The authors declare that the research was conducted in the absence of any commercial or financial relationships that could be construed as a potential conflict of interest.

## Footnotes

^1^http://dti-tk.sourceforge.net

^2^https://fsl.fmrib.ox.ac.uk

^3^http://nitrc.org/projects/spikecor

^4^http://nitrc.org/projects/phycaa_plus
