# Supplementary material for: Disturbances in Brain Physiology Due to Season Play: A Multi-Sport Study of Male and Female University Athletes
Source: Front Physiol. 2021 Mar 30;12:653603. doi: 10.3389/fphys.2021.653603 (PMC8044759; doi:10.3389/fphys.2021.653603)
Supplement: Supplementary file 1 [file Data_Sheet_1.docx]

**Supplemental File 1**: effects of season play on fractional anisotropy (FA)

As depicted in Figure S1 below, there were changes in global white matter diffusion anisotropy associated with season play. The gFA values plotted in Fig. S1A were not significantly altered at EOS (-0.71x10^-3^, [-2.19, 0.78]x10^-3^, BSR=-0.94, p=0.346) but did show a significant decline at 1MO (-1.87x10^-3^, [-3.01, -0.68]x10^-3^, BSR=-3.10, p<0.001) at an FDR of 0.05, before returning to slightly higher than baseline values at subsequent BOS2 (0.93x10^-3^, [-1.34, 3.15]x10^-3^, BSR=0.80, p=0.432). The brain maps in Fig. S1B show that modest global effects may be partly driven by spatially heterogeneous patterns of change, with longitudinal FA decreases at EOS and 1MO seen mainly within midline and frontal white matter, but FA increases seen in posterior white matter at all imaging time points.


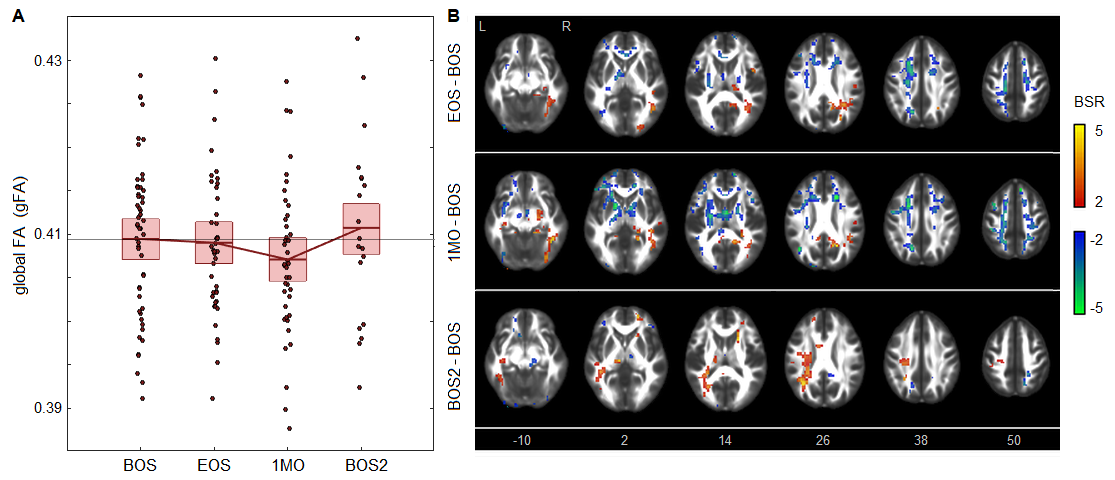


**Figure S1**: effects of season play on white matter fractional anisotropy (FA). (A) The distribution of athlete global FA (gFA) values, plotted for each imaging session. The horizontal red lines denote group means and boxes indicate 95% confidence intervals of the mean; distribution means are connected between sessions by solid red lines; the mean BOS value is also plotted as a horizontal black line as a reference. (B) Maps of regional change in FA, with relative effect sizes given as z-distributed bootstrap ratio (BSR) values, thresholded at |BSR|>2 (approximately p<0.05, uncorrected). The z-axis coordinates of axial slices in MNI space are also shown.
